# Exonic splice regulation imposes strong selection at synonymous sites

**DOI:** 10.1101/gr.233999.117

**Published:** 2018-10

**Authors:** Rosina Savisaar, Laurence D. Hurst

**Affiliations:** The Milner Centre for Evolution, Department of Biology and Biochemistry, University of Bath, Bath BA2 7AY, United Kingdom

## Abstract

What proportion of coding sequence nucleotides have roles in splicing, and how strong is the selection that maintains them? Despite a large body of research into exonic splice regulatory signals, these questions have not been answered. This is because, to our knowledge, previous investigations have not explicitly disentangled the frequency of splice regulatory elements from the strength of the evolutionary constraint under which they evolve. Current data are consistent both with a scenario of weak and diffuse constraint, enveloping large swaths of sequence, as well as with well-defined pockets of strong purifying selection. In the former case, natural selection on exonic splice enhancers (ESEs) might primarily act as a slight modifier of codon usage bias. In the latter, mutations that disrupt ESEs are likely to have large fitness and, potentially, clinical effects. To distinguish between these scenarios, we used several different methods to determine the distribution of selection coefficients for new mutations within ESEs. The analyses converged to suggest that ∼15%–20% of fourfold degenerate sites are part of functional ESEs. Most of these sites are under strong evolutionary constraint. Therefore, exonic splice regulation does not simply impose a weak bias that gently nudges coding sequence evolution in a particular direction. Rather, the selection to preserve these motifs is a strong force that severely constrains the evolution of a substantial proportion of coding nucleotides. Thus synonymous mutations that disrupt ESEs should be considered as a potentially common cause of single-locus genetic disorders.

Coding sequences (CDSs) have functions beyond coding. For example, they can harbor binding sites to transcription factors (Stergachis et al. 2013; Birnbaum et al. 2014; Blumberg et al. 2014; Reyna-Llorens et al. 2018; although see Xing and He 2015; Agoglia and Fraser 2016) or microRNAs (Lewis et al. 2005; Hurst 2006; Forman et al. 2008; Fang and Rajewski 2011; Hausser et al. 2013; Akhtar et al. 2015; Liu et al. 2015). In mammals, a prominent regulatory layer within the CDS consists of exonic splice regulatory (ESR) signals, such as exonic splice enhancers (ESEs) (Blencowe 2000; Cáceres and Hurst 2013; Lee and Rio 2015) and silencers (ESSs) (Wang et al. 2004; Lee and Rio 2015). The existence of ESR has been known for decades (Somasekhar and Mertz 1985; Reed and Maniatis 1986; Watakabe et al. 1993), and a wealth of research has accumulated showing that these elements are under purifying selection (Fairbrother et al. 2004a; Carlini and Genut 2006; Parmley et al. 2006, 2007; Ke et al. 2008; Sterne-Weiler et al. 2011; Cáceres and Hurst 2013; Ramalho et al. 2013) and that, at least in some instances, their disruption can cause disease (Lim et al. 2011; Sterne-Weiler et al. 2011; Sterne-Weiler and Sanford 2014; Supek et al. 2014; Xiong et al. 2015; Scotti and Swanson 2016; Takata et al. 2016; Wu and Hurst 2016). The conventional wisdom according to which all mammalian synonymous sites are nonfunctional and neutrally evolving has thus been put to rest.

However, despite numerous studies that have demonstrated that exonic splice regulatory elements are under purifying selection (Fairbrother et al. 2004a; Carlini and Genut 2006; Parmley et al. 2006, 2007; Ke et al. 2008; Sterne-Weiler et al. 2011; Cáceres and Hurst 2013; Ramalho et al. 2013), to our knowledge, none have attempted to determine the strength of this selection. Typically, studies measure the decrease in divergence/polymorphism rates within ESRs when compared to a putatively neutral baseline. This decrease depends both on the strength of selection and on the frequency of functional elements, without disentangling these two factors. As a result, current work is consistent both with a scenario of weak and diffuse selection, enveloping a large proportion of sequence but imposing only slight evolutionary constraint, as well as with well-localized pockets of strong selection. In the former case, selection to conserve ESRs would primarily have the effect of imposing a slight bias on codon usage. In the latter case, ESR-disrupting mutations would have large fitness effects that might frequently be clinically relevant. In determining whether, for example, a synonymous mutation within an ESE is a likely cause of a single-locus disease, it is thus important to be able to distinguish between these two possibilities.

Although we are not aware of attempts to distinguish between these two scenarios at ESRs, such studies do exist for selective constraints at synonymous sites more generally. These studies could provide an upper bound: the frequency of synonymous sites that function in splicing cannot exceed the frequency of functional synonymous sites in general. Similarly, strong selection to preserve ESRs is unlikely if none is detected at synonymous sites more broadly. Several earlier papers concluded that selection at synonymous sites was weak at best—a mere bias on the largely neutral turnover of silent sites (Lu and Wu 2005; Williamson et al. 2005; Comeron 2006; Kondrashov et al. 2006; Racimo and Schraiber 2014). Other investigators, however, have uncovered evidence for strong negative selection at 22% and 11% of synonymous sites in *Drosophila* and in human, respectively (Keightley and Halligan 2011; Lawrie et al. 2013), and have claimed that certain earlier studies were methodologically biased toward discovering weak selection alone (Lawrie et al. 2013; Machado et al. 2017). It is unknown whether or not selection on splice regulatory elements is a major contributor to this strong selection in human. In *Drosophila*, Machado et al. (2017) found that alternatively spliced genes contributed more to the signal of strong selection at synonymous sites than did constitutively spliced genes. However, this effect was dwarfed by a much greater effect of codon optimality. It is unclear what the expected pattern is in human, given that evidence for selection for codon optimality is much weaker than in *Drosophila* (Akashi 1995; Moriyama and Powell 1997; Urrutia and Hurst 2001; dos Reis et al. 2004; Lavner and Kotlar 2005; Kotlar and Lavner 2006; Waldman et al. 2010; Shabalina et al. 2013; Gingold et al. 2014).

In this paper, we determined both the frequency of functional ESEs in human CDSs as well as the strength of the selection that maintains them. We only considered fourfold degenerate sites. This removed the confound of selection on protein structure and also made our work relevant to the broader problem of selection at synonymous sites. In order to disentangle site frequency from strength of selection, we had to elucidate the distribution of fitness effects (DFE) at these positions (Eyre-Walker and Keightley 2007). The DFE specifies the distribution of selective coefficients for incoming mutations at a set of sites. Many methods exist for determining this distribution (Nielsen and Yang 2003; Piganeau and Eyre-Walker 2003; Yampolsky et al. 2005; Eyre-Walker et al. 2006; Loewe et al. 2006; Boyko et al. 2008; Keightley and Halligan 2011; Schneider et al. 2011; Wilson et al. 2011; Gronau et al. 2013; Lawrie et al. 2013; Racimo and Schraiber 2014; Keightley et al. 2016; Kim et al. 2017; Tataru et al. 2017; Barton and Zeng 2018). All of these methods come with particular caveats and can produce misleading results in certain circumstances (for instance, in the presence of positive selection or very weak negative selection). We therefore used several complementary approaches to obtain information about the DFE, so that the conclusions we drew would be supported by several different types of analysis.

## Results

### Analysis of nucleotide divergence suggests that at least 20%–30% of INT3 ESE sites are under selection

A first approach to determining the frequency of functional elements is to compare rates of synonymous substitution between ESE and control sites (Parmley et al. 2006; Cáceres and Hurst 2013; Savisaar and Hurst 2017a). The excess conservation at ESE sites is taken to reflect the proportion of motif hits that are selectively maintained and hence functional. The remainder would simply represent the chance occurrence of *k*-mers. Based on the frequency of the motifs, it is then possible to calculate the proportion of all coding nucleotides that are splice regulatory.

We determined the rate of synonymous evolution (*d*_S_; alignment to macaque) for either ESE sites or roughly nucleotide-matched control sites. Predicted ancestral CpG/GpC sites were discarded to eliminate CpG hypermutability effects (Sved and Bird 1990). To quantify constraint, we used a measure that we term *normalized d*_S_ (*normalized d*_S_ = [(*ESE d*_S_ − *control d*_S_)/*control d*_S_]), which is expected to be roughly zero under neutral evolution and below zero under purifying selection. We obtained a normalized *d*_S_ of ≈ −0.300 (ESE *d*_S_ ≈ 0.038; control *d*_S_ ≈ 0.054; one-tailed empirical *P* ∼ 0.001) (Supplemental Fig. S1; Supplemental Table S3). This initial analysis suggests that ∼30% of ESE motif occurrences are functional. Importantly, this method may overestimate conservation levels by about a third because it does not control for context-dependent mutational biases (Supplemental Text S2). A more conservative estimate would therefore put the proportion of functional ESE sites at ∼20%.

An important caveat is that we ignored ESE degeneracy. For instance, an A-to-T mutation at the last position in the ESE GAAGAA should be neutral with regards to ESE function because GAAGAT is also an ESE. Such sites might be functional, yet nevertheless accumulate substitutions, and lead us to underestimate levels of purifying selection. We thus repeated the analysis ignoring those human–macaque divergences that merely converted one ESE to another (Supplemental Text S3; Supplemental Figs. S2, S3; Supplemental Table S20). This procedure removed about a quarter of the substitutions (ESE *d*_S_ decreased from ≈0.038 to ≈0.029). However, after controlling for the number and nucleotide composition of the discarded sites (Supplemental Text S3), we found that effects on conservation were slight (control *d*_S_ ≈ 0.044, normalized *d*_S_ ∼ −0.336). We tested for an effect of degeneracy also when conducting the other analyses reported in this paper (Supplemental Text S3). In all cases, there was only a slight effect on the results. Therefore, it appears that most of the time, an ESE is not interchangeable with another ESE at the same position.

Finally, because ESEs are enriched at exon ends (Fairbrother et al. 2004a; Cáceres and Hurst 2013), it is possible that we sampled motif sites primarily from the exon end and control sites more from the exon core. If exon ends are more conserved than cores for ESE-independent reasons (Cáceres and Hurst 2013), this could artifactually lead to a signal of purifying selection on the motifs. We therefore repeated the normalized *d*_S_ analysis on sequence from the 5′ ends of exons only (first 69 base pairs [bp]). The results were similar to those obtained for full CDSs (ESE *d*_S_ ∼ 0.035; control *d*_S_ ∼ 0.051; normalized *d*_S_ ∼ −0.312), suggesting that the purifying selection we detect is not an artifact of sampling control sites from exon cores.

In conclusion, the nucleotide divergence analysis suggests that ∼20%–30% of ESE sites are functional. However, for reasons outlined in the next section, this estimate is potentially problematic.

### INSIGHT finds no evidence for positive selection and reports strong negative selection at about a quarter of ESE sites

The analysis reported in the previous section does not disentangle the frequency of selected sites from the strength and mode of selection. As a result, the estimate for site frequency could be incorrect. Specifically, if there are functional sites where the negative selection is so weak that substitutions are still observed, then this analysis may underestimate the proportion of selected sites. In addition, if the data includes fast-evolving positively selected sites (as detected for ESEs in Ke et al. 2008 and for synonymous sites more generally in Resch et al. 2007), their faster evolution might cancel out some of the signal for purifying selection. We therefore turned to more complex approaches, which allowed us to address both of these issues.

The first method used was INSIGHT (Gronau et al. 2013), which can distinguish between weak negative, strong negative, and positive selection. INSIGHT is based on the assumption that distinct selective modes affect patterns of polymorphism and divergence differently. For instance, it assumes that sites under strong negative selection display neither divergence nor polymorphisms, whereas sites under weak negative selection can harbor polymorphisms. It compares such patterns between elements of interest and control sites: in our cases, fourfold degenerate ESE positions and roughly nucleotide-matched controls. INSIGHT uses a maximum likelihood–based approach to fit three parameters: *ρ* (the fraction of sites under any kind of selection), *η* (scaling factor on the divergence rate at selected sites; expected to be zero if no positive selection is present), and γ (scaling factor on the site frequency spectrum at selected sites). Based on *η* and *γ*, INSIGHT also computes *α* (the fraction of divergences driven to fixation by positive selection) and *τ* (the fraction of polymorphisms under weak negative selection). The method cannot determine the relative proportions of sites under strong or weak negative selection, but it can indicate whether there is any evidence at all for weak negative selection.

Because INSIGHT also relies on polymorphism data, the CpG-filtering method used above may not be appropriate anymore. Earlier, we filtered out only those positions where we predicted that the human–macaque MRCA had CpG (*ancestral filtering*). However, CpGs that have arisen since the human–macaque split might also be enriched in polymorphisms. An alternative strategy is to discard positions where the human reference sequence has CpG (*human filtering*). Ancestral filtering should be better suited for primarily divergence-based INSIGHT estimates (*η* and *α*), while human filtering should be better for primarily polymorphism-based estimates (*γ* and *τ*; this is a rough heuristic—all estimates are expected to be sensitive to both divergence and polymorphism to some extent). The best approach for *ρ* is unclear.

**Figure 1.**
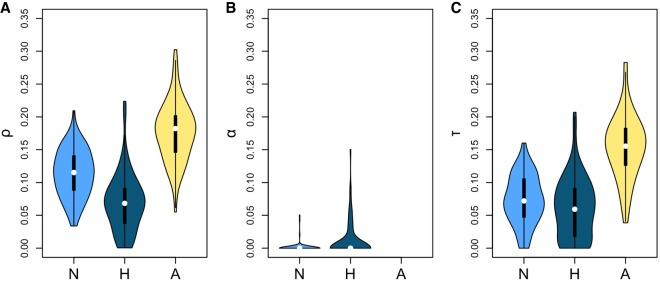
Distribution of INSIGHT statistics from 100 negative control runs using different methods of CpG-filtering. (N) No filtering; (H) human filtering; (A) ancestral filtering. Human filtering has the lowest false-positive rate for *ρ* (the fraction of sites under selection; *A*) and for *τ* (the fraction of polymorphisms under weak negative selection; *C*). Ancestral filtering performs best for *α* (the fraction of divergences driven to fixation by positive selection; *B*).

**Figure 2.**
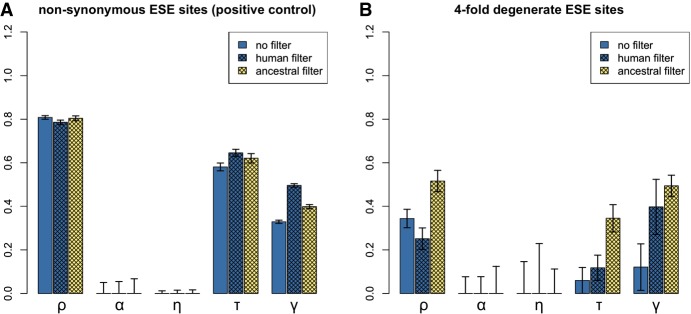
INSIGHT estimates for nonsynonymous (*A*) and for fourfold degenerate (*B*) ESE sites. (*ρ*) Fraction of sites under selection; (*α*) fraction of divergences driven to fixation by positive selection; (*η*) scaling factor on the divergence rate at selected sites; (*τ*) fraction of polymorphisms under weak negative selection; (γ) scaling factor on the site frequency spectrum at selected sites. Human CpG-filtering is expected to be more reliable for *ρ*, *τ*, and *γ*; ancestral CpG-filtering is expected to perform better for *α* and *η*. For the positive control (*A*), INSIGHT detects significant evidence for negative selection, including weak negative selection. At fourfold degenerate ESE sites, INSIGHT reports negative selection, which is likely mostly strong, as there is little to no significant evidence for weak negative selection with the more reliable human CpG-filtering method.

To test these assumptions, we performed a negative control. Over 100 simulations, we shuffled ESE and control sites within each gene and ran INSIGHT on the shuffled data (Fig. 1; Supplemental Table S4). As predicted, human filtering gave the lower false-positive rate for *τ* (Dunn-Bonferroni test *P* < 2 × 10^−16^ for comparison with ancestral filtering and 0.3 for comparison with no filtering), whereas ancestral filtering performed better for *α* (Dunn-Bonferroni test *P* ∼ 1.9 × 10^−7^ for comparison with human filtering and ∼0.231 for comparison with no filtering). In addition, human filtering had the lower false-positive rate for *ρ* (Dunn-Bonferroni test *P* < 2 × 10^−16^ for comparison with ancestral filtering and ∼7.4 × 10^−8^ for comparison with no filtering). Additionally, we carried out a positive control using nonsynonymous sites. As expected, INSIGHT detected a mixture of strong and weak selection to preserve protein structure (Fig. 2A; Supplemental Text S4).

We next ran the analysis on fourfold degenerate ESE positions (Fig. 2B; Supplemental Table S5). Both CpG-filtering methods agreed that there was no evidence for positive selection. Ancestral filtering predicted a higher percentage of sites under selection than human filtering (∼51.6% vs. ∼25.1%; empirical *P*-values ∼0.010 for both). This might be due to it detecting greater levels of weak negative selection (percentage of polymorphisms under weak negative selection ∼34.5% vs. ∼11.8%; empirical one-tailed *P*-values for decreased allele frequencies at selected sites ∼0.010 vs. ∼0.079, respectively). Indeed, when INSIGHT was run with the assumption of no weak negative selection (γ fixed at zero), ancestral filtering returned an estimate for the percentage of selected sites that was nearly identical to that obtained with human filtering (∼25.9%). Given that in our negative control, ancestral filtering had a tendency to overestimate levels of weak negative selection (Fig. 1), we consider that the human filtering results are more likely to be accurate. We also considered the possibility that the differences between ancestral and human filtering results could be due to the use of different sets of genes; however, further analysis revealed this to be unlikely (Supplemental Text S4).

In conclusion, INSIGHT found no evidence for positive selection at ESE sites. This suggests that the normalized *d*_S_ statistic determined in the previous section reflects the effects of negative selection alone. In addition, about a quarter of ESE sites were found to be under selection. This is fairly close to the results obtained above using divergence data alone. Most, if not all, of these sites appear to be under strong negative selection, suggesting that mutations within functional ESEs are likely to have large fitness, and potentially clinical, effects. However, such inferences regarding the shape of the negative DFE must be treated with caution. This is because the INSIGHT model also assumes that even weak negative selection must be strong enough to effectively preclude substitutions, which, as discussed above, is not necessarily the case. We might therefore still be underestimating the proportion of functional sites. We therefore next turned to methods based on polymorphism data alone, so as not to make assumptions as to the effects of very weak negative selection on nucleotide divergence.

### Polymorphism data suggest that the primary mode of selection is strong and constrains about a third of ESE sites

Polymorphism data contain two primary kinds of information on negative selection. First, mutations that are strongly selected against rarely spread beyond very low frequencies in the population and are frequently lost. They are thus less likely to be sampled. Lower than expected numbers of segregating sites are therefore a signal of strong negative selection. With weak negative selection, mutations can spread further in the population, but MAFs are decreased compared to expectation. We devised two tests based on this reasoning. The first was to compare the proportion of polymorphic sites at ESE sites and at control sites using a *χ*^2^ test. The second was to compare MAFs of segregating sites between ESE and control sites using a one-tailed Mann-Whitney *U* test. Because these methods are based on polymorphism data alone, the preferred method for removing CpG sites should be human filtering (although, as can be seen in Table 1, ancestral filtering gives qualitatively similar results).

**Table 1.**
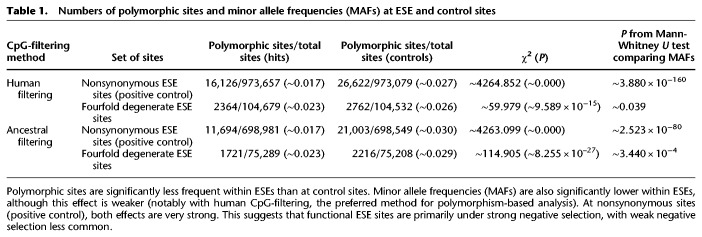
Numbers of polymorphic sites and minor allele frequencies (MAFs) at ESE and control sites

As a positive control, we performed both tests on nonsynonymous ESE sites (with fourfold degenerate ESE sites as controls), as in the previous section. This analysis should provide strong evidence for both strong and weak negative selection. Indeed, both the proportion of polymorphic sites as well as minor allele frequencies (MAFs) were significantly decreased (Table 1). We then performed the analysis on fourfold degenerate ESE sites. We found a significant decrease in both the fraction of polymorphic sites as well as in MAFs when comparing ESEs to control sites (Table 1). However, the effect was much more significant for the fraction of polymorphic sites, suggesting that the primary mode of selection is strong. These results are consistent with the INSIGHT results reported above, which also suggest that although a minority of sites may be under weak negative selection, the primary mode of selection is strong. The fact that the comparison of MAFs and INSIGHT lead to such similar results suggests that the weak negative selection that is reported is unlikely to be sufficiently weak to allow for substitutions, as selection this weak should only be detected by the former.

In order to turn these qualitative statements into population-scaled selective coefficient (*N_e_s*) estimates, we used the multiDFE method developed by Kousathanas and Keightley (2013) (an expansion of the older DFEalpha program [Keightley and Eyre-Walker 2007]). multiDFE uses Fisher-Wright transition matrix methods to generate the expected allele frequencies in a population under the assumption that incoming mutations are sampled from a particular distribution of selective coefficients. A maximum likelihood–based procedure then fits the parameters of the distribution so as to minimize the discrepancy between this allele frequency vector and the true site frequency spectrum observed in the population.

Many different computational methods exist for determining the DFE (Nielsen and Yang 2003; Piganeau and Eyre-Walker 2003; Yampolsky et al. 2005; Eyre-Walker et al. 2006; Loewe et al. 2006; Boyko et al. 2008; Keightley and Halligan 2011; Schneider et al. 2011; Wilson et al. 2011; Lawrie et al. 2013; Racimo and Schraiber 2014; Keightley et al. 2016; Kim et al. 2017; Tataru et al. 2017; Barton and Zeng 2018). We have chosen to use multiDFE in particular for three reasons. First, unlike methods that assume a particular kind of distribution beforehand (e.g., Piganeau and Eyre-Walker 2003; Keightley and Eyre-Walker 2007; Tataru et al. 2017), one can pick the best fit from many different types of distributions, including nonparametric ones. This is important because if the true distribution is substantially different than the distribution that is being assumed, this can lead to erroneous conclusions regarding the properties of the DFE (Kousathanas and Keightley 2013). Second, the method explicitly accounts for population change in the past. Although accounting for demography has become commonplace (Eyre-Walker et al. 2006; Boyko et al. 2008; Lawrie et al. 2013; Kim et al. 2017; Tataru et al. 2017), multiDFE (and earlier iterations of the method [Keightley and Eyre-Walker 2007]) has the particularity of estimating demographic and selection parameters simultaneously. Third, multiDFE (like its predecessor [Keightley and Eyre-Walker 2007]) considers not only the frequency spectrum of segregating sites but also the proportion of monomorphic sites, enabling it to more readily detect strong negative selection (similarly to Lawrie et al. 2013). To our knowledge, no other method has all three of these properties (with the potential exception of that of Kim et al. 2017).

When interpreting the multiDFE output, we followed Yampolsky et al. (2005). We assumed that mutations were *effectively neutral* if the associated population-scaled selective coefficient (*N_e_s*) was below ∼0.25. We also assumed that the probability of fixation becomes negligible above ∼2*N_e_s* (*weak negative selection*), while contribution to polymorphism becomes negligible above ∼10*N_e_s* (*strong negative selection*). Mutations that are under negative selection that is too weak to prevent fixation should therefore be associated to a *N_e_s* value between ∼0.25 and ∼2 (*very weak negative selection*). Note that because positive selection was not considered, we only used positive *N_e_s* values, with increasing *N_e_s* signifying increasingly strong negative selection. Importantly, multiDFE returns the distribution of selective coefficients for incoming mutations, not for sites. However, given that ESE degeneracy seems to be rare, we ignored this distinction and considered the proportion of incoming mutations that are under selection as a proxy for the frequency of functional sites. We limited the polymorphism data used to the Yoruban subsample of the 1000 Genomes data set, as methods such as multiDFE are susceptible to become unreliable with large samples (Kim et al. 2017).

**Figure 3.**
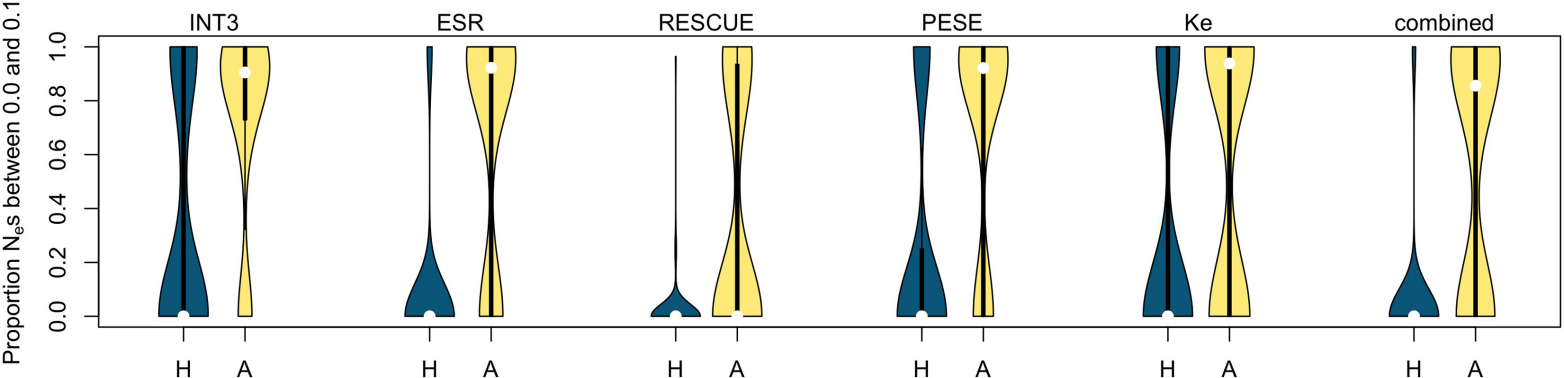
Negative control. Proportion of *N_e_s* below 0.1 over 100 control simulations, for the different motif sets and for human (H) and ancestral (A) CpG-filtering. Ancestral filtering consistently delivers a lower false-positive rate (less density at the bottom).

Regarding the handling of CpGs, human filtering was expected to be the most appropriate because multiDFE is purely polymorphism based. We nevertheless performed 100 negative control runs to determine the false-positive rate for the two methods (Supplemental Text S5; Supplemental Table S7). Ancestral CpG-filtering achieved a substantially lower false-positive rate than human filtering. With human filtering, only 15% of runs exhibited >80% of the density below *N_e_s* = 0.1, whereas this percentage was 71% for the ancestral filtering (Fig. 3, leftmost plot).

**Figure 4.**
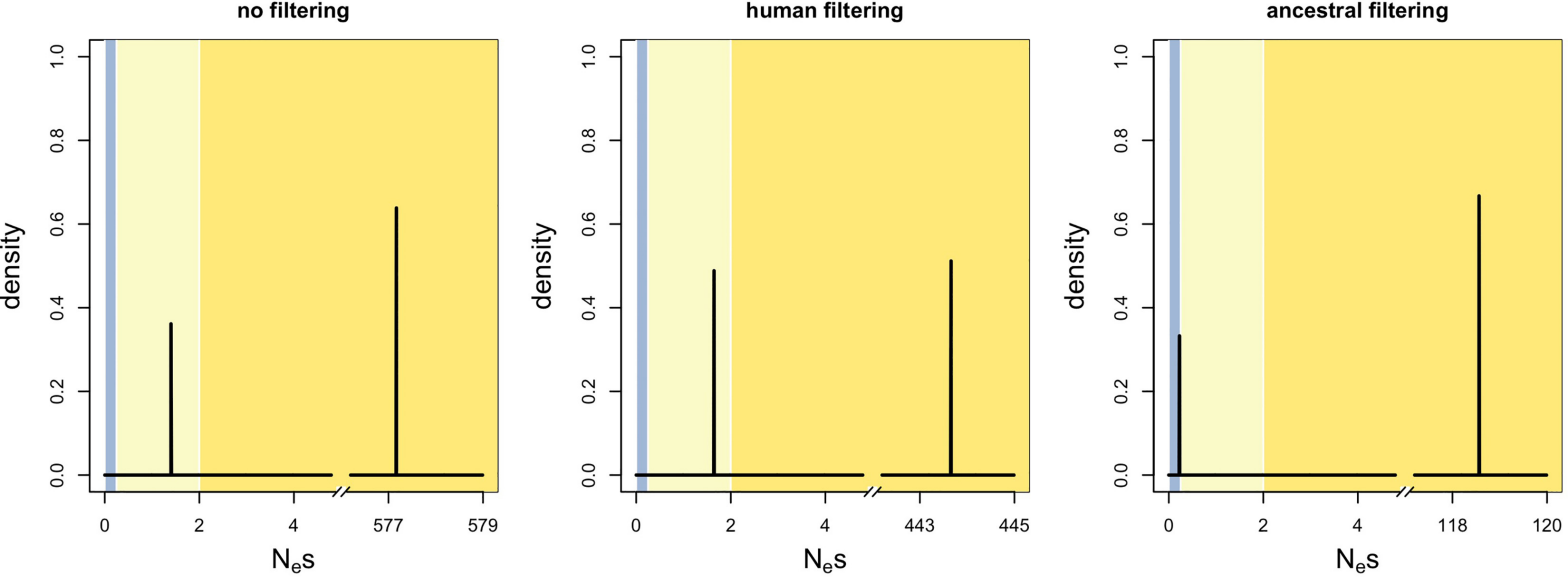
Positive control. Distribution of fitness effects (DFE) for non-fourfold degenerate ESE sites, using fourfold degenerate non-ESE sites as control. (Blue zone) Effective neutrality; (light yellow zone) very weak negative selection; (dark yellow zone) weak to strong negative selection. “No filtering,” “human filtering,” and “ancestral filtering” refer to different methods for filtering out CpG dinucleotides (for more details, see main text).

Because of these results, we largely relied on the ancestral filtering method when drawing conclusions from the multiDFE analysis. The negative control also demonstrated the drastic importance of accounting for demographic history, as ignoring it led to a large increase in the false-positive rate (for details, see Supplemental Text S5 and Supplemental Fig. S4). As with INSIGHT, we also performed a positive control by analyzing non-fourfold degenerate ESE sites (Fig. 4; Supplemental Text S5). Our protocol for running multiDFE appears capable of detecting both strong and weak negative selection.

**Figure 5.**
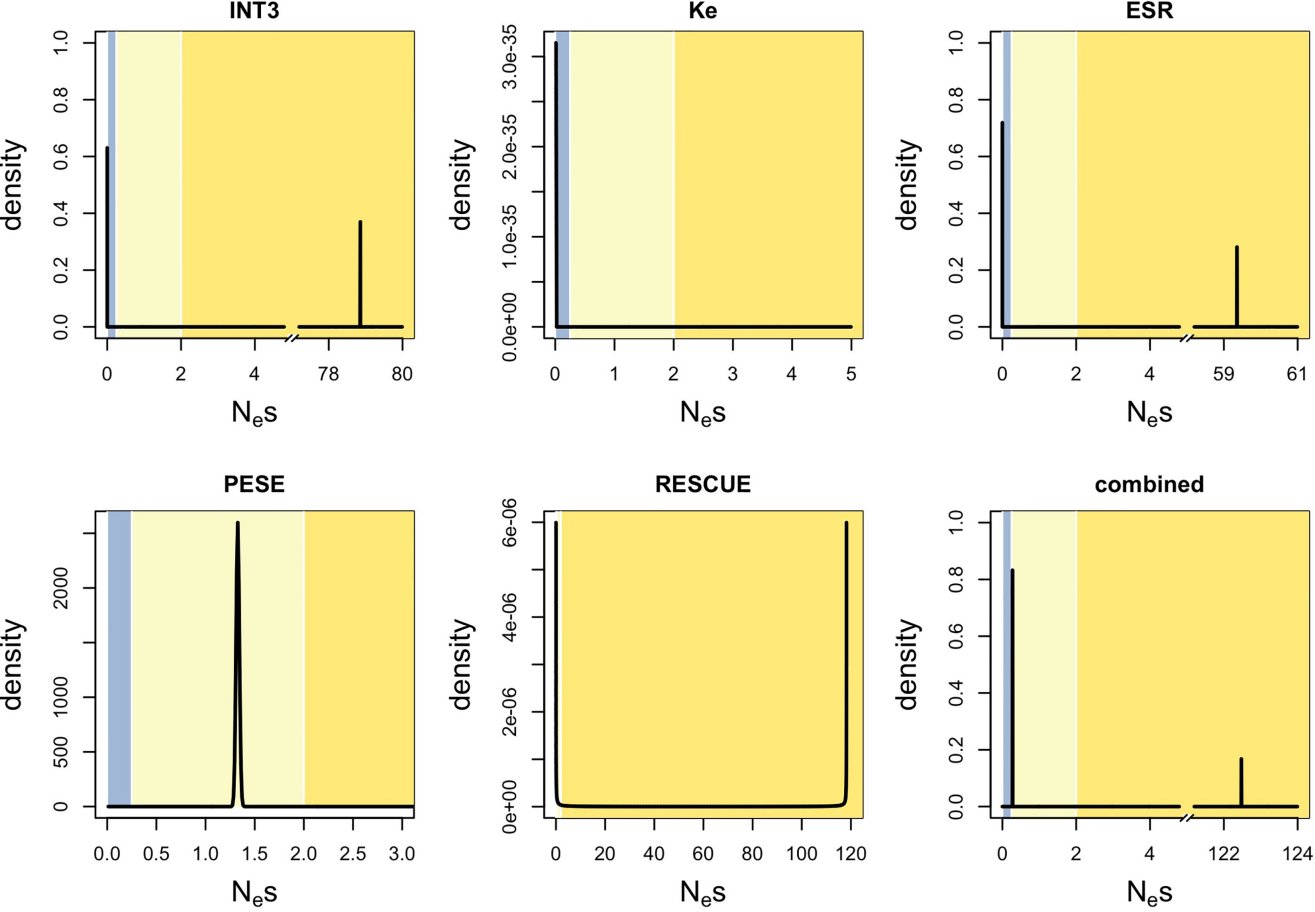
DFE for incoming mutations at ESE sites, using different motif sets. Ancestral CpG-filtering was used in all cases. (Blue zone) Effective neutrality; (light yellow zone) very weak negative selection; (dark yellow zone) weak to strong negative selection. Note that although the distribution obtained for RESCUE looks different from that obtained for INT3 or ESR, all three convey very similar information (the majority of mutations within effective neutrality and a minority within strong negative selection). The visual difference is due to the fact that for RESCUE, the best fit was a beta distribution, whereas for the other two it was a two-spikes distribution.

We then ran multiDFE on fourfold degenerate ESE sites with nucleotide-matched fourfold degenerate non-ESE sites as control. The best-fit model with ancestral filtering was a two-spikes model with population size change in the past. multiDFE indicated that ∼63% of the density lay within effective neutrality and that ∼37% of mutations were under strong negative selection (with *N_e_s* ∼ 100) (Fig. 5, top left plot; Supplemental Table S8; Supplemental Text S5 for other CpG-filtering methods). multiDFE thus provided no evidence for substantial levels of very weak negative selection at fourfold degenerate ESE positions: Mutations are either effectively neutral (about two-thirds) or sufficiently deleterious that their fixation is extremely unlikely (about one-third). We also detected no density between *N_e_s* 2 and 10. This would suggest that deleterious mutations are under such strong selection that polymorphisms can only occur at very low frequencies and are thus unlikely to be sampled. This is inconsistent with the INSIGHT results, as well as the comparison of MAFs performed above, which provided (near-)significant evidence for weak negative selection. This could be explained by the use of a smaller sample for the polymorphism data, which can lead to overestimation of the prevalence of strong negative selection (Kim et al. 2017) at the detriment of weaker selection.

To conclude, the various analyses converge to suggest that about a quarter to a third of fourfold degenerate INT3 ESE positions are functional, while the remainder are primarily noise—nothing more than hexamers occurring by chance, or perhaps sites within functional hexamers that are themselves not under selection. Given the decrease in MAFs within ESEs, it is possible that some very weakly negatively selected sites are present, which would increase the proportion of functional sites. However, any such increase is unlikely to be substantial because of the weakness of the evidence for weak negative selection and because of the similarity between the results obtained using INSIGHT and in the MAF comparison test. If our failure to control for contextual mutation biases indeed leads to an overestimation of about a third (as suggested in the first section of the Results), the functional fraction decreases to about a fifth to a quarter of the sites. Given that ∼18% of coding nucleotides are part of an ESE, this would mean that overall, ∼4%–6% of fourfold degenerate sites are constrained because of the need to preserve INT3 motifs. However, the selection acting at most of these sites would be strong, and mutations are thus likely to have phenotypic and clinical relevance.

### Expanding the motif set

The above analysis comes with a major caveat. INT3, the set of ESE motifs used, was crafted to be particularly conservative (Cáceres and Hurst 2013) and is thus probably enriched for a core set of particularly constrained ESE motifs. Do our results apply to ESEs more generally? INT3 is composed of motifs that appeared in at least three out of four previously published sets of ESEs (Cáceres and Hurst 2013). We expanded our set by combining motifs from all four sets. These motif sets will be referred to as ESR (Goren et al. 2006), PESE (Zhang and Chasin 2004), RESCUE (Fairbrother et al. 2002; Fairbrother et al. 2004b), and Ke (Ke et al. 2011) (for Ke, only the 400 motifs with the strongest evidence for being an ESE were considered). We refer the reader to Cáceres and Hurst (2013) for more information on the origin and properties of each set.

**Figure 6.**
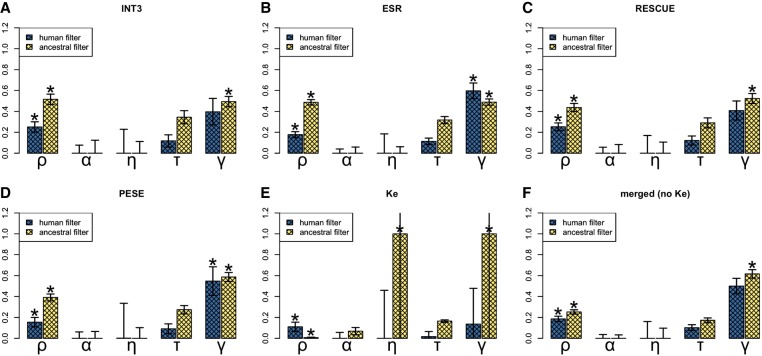
INSIGHT estimates for different sets of ESE motifs. (*A*) INT3; (*B*) ESR; (*C*) RESCUE; (*D*) PESE; (*E*) Ke; and (*F*) all sets combined (except for Ke). (*ρ*) Fraction of sites under selection; (*α*) fraction of divergences driven to fixation by positive selection; (*η*) scaling factor on the divergence rate at selected sites; (*τ*) fraction of polymorphisms under weak negative selection; (γ) scaling factor on the site frequency spectrum at selected sites. Human CpG-filtering is expected to be more reliable for *ρ*, *τ*, and γ; ancestral CpG-filtering is expected to perform better for *α* and *η*. An asterisk (*) indicates an empirical one-tailed *P*-value of <0.05. There are no *P*-values for *α* and *τ*. NB: For visualization purposes, the *η* and *γ* values for Ke (ancestral CpG-filtering) have been capped at one. The true values are *η* ≈ 10.625 and *γ* ≈ 28.543.

Before taking the union of the sets, it was important to verify that none showed signs of positive selection, as this could mislead the majority of the analyses we perform (Tataru et al. 2017). We therefore ran INSIGHT on each of the motif sets. The only data set to show evidence for positive selection was Ke (Fig. 6; Supplemental Table S15). This is consistent with the results from Cáceres and Hurst (2013), where this set also exhibited unusual properties: It was fast- rather than slow-evolving, and it was enriched in exon cores over exon flanks. To prevent the positive selection from misleading any further analyses, we constructed the combined set of motifs by merging all sets except for Ke. This resulted in a final combined set of 2528 unique motifs (468 hexamers and 2060 octamers).

The three sets other than Ke, including the combined set, behaved fairly similarly to INT3 (Fig. 6). None showed evidence for positive selection. This contradicts the results of Ke et al. (2008), who reported positive selection on PESE and RESCUE motifs. However, Ke et al. (2008) studied constitutively and alternatively spliced exons separately and only found positive selection for the former. As we are not making this distinction, any signs of positive selection present in constitutively spliced exons alone might be undetectable in the overall signal. The percentage of sites under selection was estimated to be between ∼15% and ∼25% for all sets, with the combined set at ∼18.6%. Only ESR and PESE showed significant evidence for weak negative selection. None was observed for the combined set, suggesting that although some ESEs might indeed be under weak negative selection, this is a minority. Contrary to our expectations, the INT3 set is therefore not exceptional in its evolutionary properties. The effect of merging the sets appears to be one of simply including more sites rather than of qualitatively altering the DFE. Note that just like above for INT3 alone, we are basing our conclusions on human CpG-filtering for *ρ*, *γ*, and *τ* and on ancestral filtering for *η* and *α*. This is because this approach is expected to lead to the lowest false-positive rate for all sets (Supplemental Tables S10–S14).

How does considering a larger set of ESEs affect our estimate for normalized *d*_S_? We calculated normalized *d*_S_ for each of the sets. The results were again consistent between sets, with normalized *d*_*S*_ values between −0.3 and −0.2 (Table 2; for negative control, see Supplemental Table S9). Ke was once again an outlier, as it exhibited a positive normalized *d*_S_ of ∼0.148, consistent with widespread positive selection. We considered the possibility that this signal of fast evolution could be due to sampling ESE and control sites from different parts of the exon. Alternatively, it could be driven by unusual Ke motifs that have a stronger splice enhancer effect when placed in the exon core. However, we found support for neither of these hypotheses (Supplemental Text S6).

**Table 2.**
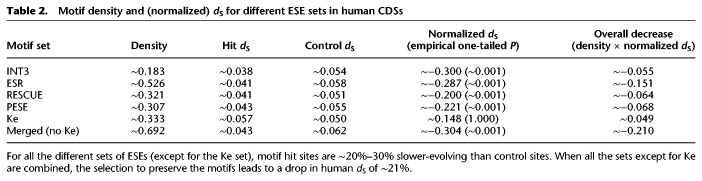
Motif density and (normalized) *d*_S_ for different ESE sets in human CDSs

With the combined set, we obtained a motif density of ∼0.692 and a normalized *d*_S_ of ∼ −0.304. If we consider that our estimate is potentially inflated because of context-dependent mutational biases (Supplemental Text S2), we can conclude that ESE preservation causes a decrease of ∼15%–20% in overall human *d*_S_. Thus, one in five fourfold degenerate bases overlap with a functional ESE. This conclusion is not qualitatively altered by the exclusion of the ESR set, which was defined partially based on motif conservation (Supplemental Text S6). Therefore, our initial conclusion of functional ESEs being rare was greatly biased by our use of a set of motifs with a low false-positive rate but a high false-negative rate.

We next performed the polymorphism-based analyses to check for very weak negative selection. All sets displayed significantly decreased polymorphism frequencies. All of the individual sets, except for ESR and Ke, also showed a significant decrease in MAF. However, this was not the case for the combined set, and for all sets, the evidence for decreased MAF was considerably weaker than the evidence for decreased polymorphism rates (Table 3). This supports the earlier conclusion that the primary mode of selection at ESE sites is strong negative selection. We note that the Ke set also displayed decreased polymorphism frequencies. However, given the normalized *d*_S_ and INSIGHT results, it is likely that this reflects reduced variation caused by recent fixation of positively selected variants more so than purifying selection.

**Table 3.**
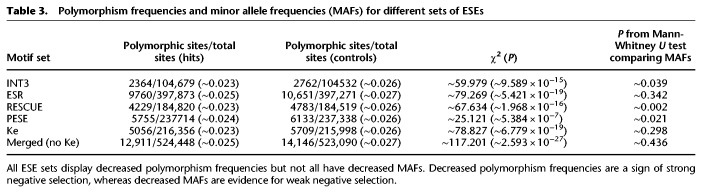
Polymorphism frequencies and minor allele frequencies (MAFs) for different sets of ESEs

Finally, we performed the multiDFE analysis (Fig. 5; Supplemental Table S18). We only used ancestral CpG-filtering, as the negative control demonstrated this method to have the lower false-positive rate for all sets (Fig. 3; Supplemental Tables S16, S17). ESR and RESCUE behaved similarly to INT3: multiDFE predicted a two-spike distribution of selective coefficients with a smaller peak (∼20%–30% of the density) within the zone of strong negative selection and a larger peak within effective neutrality. No purifying selection was detected for the Ke data set. The results for PESE were more surprising: multiDFE located all of the density within a single peak just below *N_e_s* = 1.5. This would suggest that all PESE motif hits are under very weak negative selection. This scenario is clearly unrealistic. It is also inconsistent with the results from the other analyses: Both INSIGHT and polymorphism frequencies indicated the presence of strong negative selection. We noticed during the preparation of this paper that when presented with a large set of motifs that is likely to be heterogeneous in terms of the selective modes involved (the RBP motifs data set from Savisaar and Hurst 2017a), multiDFE was unable to tease apart the different selective modes and predicted a single peak within the region of very weak negative selection (Supplemental Table S19). It is possible that a similar situation is occurring with PESE. Note that PESE is the only set that is composed of octamers rather than hexamers. This peculiarity, however, does not explain the multiDFE results: When we compiled a new motif set out of all those hexamers that appeared at least seven times in the PESE set (akin to the procedure used in Cáceres and Hurst 2013) and ran multiDFE on the resulting hit sites, the best-fit DFEs were qualitatively similar to those obtained with the octamers (Supplemental Table S18).

The best-fit model for the combined set was also a two-spikes distribution with ∼16.8% of the density at a high *N_e_s* value of ∼122.474 and the remainder at ∼0.275—just above our fairly arbitrary cut-off between effective neutrality and very weak negative selection at 0.25. Taken at face value, this result would indicate that there are no neutrally evolving ESE sites. Given the rest of the results presented in this paper, this seems unlikely. In the negative control, our setup of multiDFE mistook effective neutrality for very weak negative selection on about a third of the runs (Supplemental Tables S7, S16). This could also be happening here. Alternatively, the first peak could be an amalgam of effective neutrality and very weak negative selection. However, the three-spikes model did not tease this single peak apart either—it placed a first spike with ∼57.5% of the density at *N_e_s* ∼ 0.250 and a second spike with ∼16.6% of the density at *N_e_s* ∼ 0.360. In addition, this signal for very weak negative selection appears to be due entirely to the inclusion of the PESE set. When this set was excluded, the combined set displayed a large peak (∼68.7%) within effective neutrality (*N_e_s* ∼ 3.124 × 10^−12^) and the remainder of the density within strong negative selection (*N_e_s* ∼ 58.572). Given that multiDFE does not seem to be able to tease apart the different selective modes acting on the PESE motifs, the inclusion of this set might also be introducing noise into the analysis of the full set.

## Discussion

### Strong selection on ESEs

It has been known for over a decade that human ESEs are under purifying selection (Fairbrother et al. 2004a). In this work, we have, for the first time, determined the strength of this selection. We have found that most functional ESE sites are under strong purifying selection (50 < *N_e_s* < 150). There were also indications in our data that certain sites might be under weak negative selection. We are not able to estimate how frequent such sites may be. However, considering the results as a whole, it seems likely that this is a minority. Moreover, the strong selection that we detect concerns a considerable proportion of CDS: ∼15%–20% of fourfold degenerate bases are part of a functional ESE (this percentage might be slightly higher if some of the weak negative selection is weak enough to allow for substitutions). Therefore, ESE-related selection should not be conceptualized as merely a weak bias, a gentle nudging of codon usage. Rather, it severely constrains CDS evolution. This helps explain why ESEs seem to leave such strong footprints on sequence evolution. For instance, many species show clear ESE-associated biases in nucleotide and amino acid composition toward exon ends (Parmley and Hurst 2007; Parmley et al. 2007; Warnecke et al. 2008), and the proportion of CDS close to exon ends (within 70 bp) is a strong predictor of protein evolution rates, of the same magnitude as expression level (Parmley et al. 2007).

Importantly, our results imply that mutations that disrupt ESEs usually have large fitness consequences and might therefore often be pathogenic. This conclusion is coherent with a large body of work showing a link between disruption of splice regulatory elements and disease (Sterne-Weiler and Sanford 2014; Supek et al. 2014; Xiong et al. 2015; Scotti and Swanson 2016; Takata et al. 2016; Livingstone et al. 2017; Soemedi et al. 2017). Indeed, known pathogenic SNPS are strongly enriched at exon ends where ESEs are enriched (Wu and Hurst 2016).

### Bridging experimental and evolutionary estimates for the prevalence of exonic splice information

This work was motivated by a discrepancy between results from genome-wide studies of ESE conservation and mutagenesis studies of model exons. The former have found only a few percent of CDS to be functional ESE, whereas the latter have returned estimates for the frequency of splice information ranging from about a fifth to nearly the entirety of the sequence. We discussed this problem at length in the work of Savisaar and Hurst (2017b). Among many other factors, we noted that the conservation-based estimates could not be used to reliably determine the frequency of functional sites without knowing the DFE for incoming mutations. In this publication, we have elucidated the ESE DFE and thereby produced an estimate for the frequency of functional ESE sites (15%–20% of all fourfold degenerate sites) that is in line with the lower experimentally derived figures. However, it was not the elucidation of the DFE that allowed for the gap to be bridged, as most of the selection acting at ESE sites is strong and would largely preclude substitutions. Rather, the determining factor was that we expanded the set of motifs analyzed. Therefore, an important reason why previous conservation-based estimates were so low is that small and conservative sets of motifs were considered. The gap between experimental and conservation-based estimates decreases even further if one also accounts for the fact that the experimental studies used short exons and thus enriched for exon end nucleotides, which are particularly dense in splice information (Savisaar and Hurst 2017b).

Nonetheless, it is likely that even the union set does not include all functional ESE motifs. Moreover, not all of the ESR information in the exon can be captured simply via *k*-mer searching with no regard to factors like the pre-mRNA secondary structure (Li et al. 2010) or interactions between splicing, chromatin, and the process of transcription (Gomez Acuna et al. 2013; de Almeida and Carmo-Fonseca 2014). However, the frequency of selected ESE sites cannot exceed the frequency of selected synonymous sites overall, and by most estimates, this is about a quarter of sites at most (Eory et al. 2010; Pollard et al. 2010; Keightley and Halligan 2011; though see Racimo and Schraiber 2014 and Price and Graur 2016). It is therefore likely that we are indeed capturing most exonic splice information. Note, however, that the selection acting on ESEs also includes selection for the splicing-independent roles of these motifs (Pozzoli et al. 2004; Sanford et al. 2004; Taniguchi et al. 2007; Maslon et al. 2014; Bradley et al. 2015; Savisaar and Hurst 2016). It is unclear what proportion of the functional ESE sites that we observe is maintained for splicing-independent reasons, but our earlier work suggests that it might be substantial (Savisaar and Hurst 2016).

### Selection on ESEs in the broader context of selection at synonymous sites

The DFE that we have obtained for ESEs resembles the DFE obtained for synonymous sites more generally. Both in human (Keightley and Halligan 2011) and in *Drosophila* (Lawrie et al. 2013), previous studies have reported synonymous sites to be made up of a large class of neutrally evolving sites and a smaller class of strongly selected sites. The strongly selected proportion was reported as ∼11% in human by Keightley and Halligan (2011) and as ∼22% in *Drosophila* by Lawrie et al. (2013), although in *Drosophila*, a subsequent higher-powered study also found evidence for a third class of sites that are under weak negative selection (Machado et al. 2017). Our estimate for the frequency of strongly selected ESE sites is close to the ∼11% estimate for the frequency of strongly selected synonymous sites overall (more precisely, our estimate is higher, likely because of methodological differences between the studies) (Keightley and Halligan 2011). This may suggest that the majority of synonymous constraint in humans can be accounted for by accounting for selection on ESEs.

### A very unusual subset of ESEs appears to be under positive selection

A striking finding from the present work is that a particular subset of ESEs (Ke et al. 2011) appears to be under vastly different evolutionary pressures compared to the others. This data set was among those analyzed by Cáceres and Hurst (2013) and found to differ from the other motif sets. It was fast- rather than slow-evolving and enriched in exon cores rather than flanks. Here, we have found further evidence that this ESE set, unlike the others considered, is under positive selection. It is not surprising that there might be a distinct class of ESEs that differs from others in its distribution along exons and in nucleotide composition. It is unclear, however, why these properties should correlate with positive selection. Note that the original Ke et al. (2011) paper did report both higher species conservation and lower polymorphism frequencies within their set of splice enhancers. Of these findings, we could only replicate the latter. However, given that the rest of our results, as well as those obtained by Cáceres and Hurst (2013), are more consistent with positive than with negative selection, we interpret the polymorphism depletion in the set as reflecting recent fixation events due to positive selection.

An interesting problem is whether this positive selection is primarily to gain new motifs where there were none before or simply to swap one motif for another. We have performed a preliminary analysis where we repeated the normalized *d*_S_ analysis with these motifs but only considered substitutions that turned a motif into a nonmotif (using the same methodology as in Supplemental Text S3). If the selection is to gain motifs, normalized *d*_S_ should increase. If it is to change one motif for another, it should decrease. The answer is the former: Normalized *d*_S_ sharply increased from ∼0.148 to ∼0.296. It therefore appears that our CDSs are under selection to gain new ESE motifs from the set defined by Ke et al. (2011). The reasons for this selective pressure remain to be elucidated.

## Methods

### General

Almost all analysis was conducted with the help of custom Python 3.4.2. scripts (code available at https://github.com/rosinaSav/DFE_paper_repo; last accessed June 5, 2018) using standard libraries, NumPy 1.9.1., (van der Walt et al. 2011) and Biopython 1.64 (Cock et al. 2009). Perl v5.22.2 scripts were used for interacting with the Ensembl API. R version 3.2.1. (R Core Team 2013) was used for plotting and for certain statistical tests. BEDTools 2.19.1 (Quinlan and Hall 2010) was used for operations on sequence coordinates.

### Sequences and motif searching

The analysis was performed on a high-quality filtered subset of all GRCh38 CDSs (Ensembl release 85) (Cunningham et al. 2015). The filtering procedure, as well as the subsequent clustering into paralogous families, was identical to that used by Savisaar and Hurst (2016). Our final set contained 10879 genes, which clustered into 6101 families, 1229 of which nonsingleton. The macaque sequences used for calculating normalized *d*_S_ (for more details, see main text) were derived from assembly MMUL_1, with the annotations from Ensembl release 85. The orthology relations were also obtained from the Ensembl database (for further details on the alignment procedure, see Savisaar and Hurst 2016). Supplemental Text S1 contains details on the extraction of 5′ flank and core exonic regions. The ESE set used was the INT3 intersection set described by Cáceres and Hurst (2013). The other motif sets used were obtained similarly to Cáceres and Hurst (2013). For Ke motifs (set of ESE motifs obtained in Ke et al. 2011), only the 400 showing the strongest evidence for splice enhancer activity were retained. In order to determine which Ke motifs were more active in the exon core than the flank, we retrieved Supplemental Table S1 from Ke et al. (2011) and compared the LEIsc score obtained in context HA to that obtained in context HM. Only motifs where the former was at least 0.5 units greater than the latter were retained.

### Controlling for nucleotide composition

Full details can be found in Supplemental Text S1. Briefly, within each gene, we picked a set of fourfold degenerate control sites that matched the nucleotide composition of fourfold degenerate ESE sites. For simple comparisons of nucleotide divergence (normalized *d*_S_), and for comparisons of polymorphism frequencies and MAFs, we obtained an exact match by sampling with replacement to match each ESE site with a control site of the same nucleotide composition. For the other analyses, the match was approximate, as for these methods, it was preferable to obtain more control sites than ESE sites. We therefore used an optimization method to obtain both a close match in terms of nucleotide composition and to maximize the number of control sites (for details on the control sites, see Supplemental Tables S1, S2).

### Filtering out CpG sites

We used one of three strategies for handling CpG-dinucleotides. The first strategy (no filtering) was to do no CpG-based filtering at all. The second strategy (human filtering) was to remove those sites that overlapped with a CpG/GpC dinucleotide in the human reference sequence. The third strategy (ancestral filtering) was to remove those sites that overlapped with a probable CpG/GpC dinucleotide in the human–macaque most recent common ancestor (MRCA; note that when filtering CpGs for the INSIGHT analysis, the human–chimpanzee MRCA was used instead).

Concretely, for the third strategy, we used a custom Perl script to fetch the eight-primate EPO multiple sequence alignment for our CDSs from a local installation of release 85 of the Ensembl Compara (Herrero et al. 2016) database and API. Only CDSs where a full alignment for the whole CDS and for all eight species could be obtained were kept. We then used the phyloFit program (Siepel and Haussler 2004; Hubisz et al. 2011) to calculate the posterior probabilities for the dinucleotides present at the sites in our sequences in the human–macaque MRCA (with the flag *--scale-only* turned on, with *subst-mod* set to *U2S* and *init-model* downloaded from ftp://hgdownload.cse.ucsc.edu/goldenPath/hg38/phastCons100way/hg38.phastCons100way.mod; last accessed February 14, 2017). Sites where the posterior probability of overlapping with CpG/GpC exceeded 0.5 were removed.

### Polymorphism data

Tabix version 0.2.5 (r1005) (Li 2011) was used to obtain 1000 Genomes Phase 3 data for genomic regions overlapping our CDSs (files for the different chromosomes obtained from ftp://ftp.1000genomes.ebi.ac.uk/vol1/ftp/release/20130502/ALL.chrN.phase3_shapeit2_mvncall_integrated_v3plus_nounphased.rsID.genotypes.GRCh38_dbSNP_no_SVs.vcf.gz; last accessed April 19, 2017). VCFtools 0.1.15 (Danecek et al. 2011) was used to subset so as to only keep data on Yoruban individuals for the multiDFE analysis (panel file from ftp://ftp.1000genomes.ebi.ac.uk/vol1/ftp/release/20130502/integrated_call_samples_v3.20130502.ALL.panel; last accessed April 20, 2017). Positions with polymorphisms other than biallelic SNPs or where the reported ancestral allele differed from the base in the reference genome at that position were filtered out and not used in any fully or partially polymorphism-based analyses.

### Normalized *d*_S_

Normalized *d*_S_ was calculated similarly to Savisaar and Hurst (2016). Control sites were processed similarly. In order to obtain a *P*-value, we performed 1000 simulations where each time we shuffled the motif hit and control positions within each gene (preserving the number of motif and control positions), repeated the analysis with the shuffled positions, and used the resulting distribution of normalized *d*_S_ values to calculate an empirical *P*-value (*p* = [(*n* + 1)/(*m* + 1)], where *n* is the number of simulants presenting a normalized *d*_S_ as low as or lower than the true value and *m* is the number of simulants). This simulation is expected to eliminate systematic differences in selective constraint between hits and controls. Indeed, the simulated normalized *d*_S_ values clustered symmetrically around zero (Supplemental Fig. S1; Supplemental Table S3).

### INSIGHT analysis

We used the same set of CDSs for the INSIGHT analysis as for the normalized *d*_S_ analysis but excluded sequences on the sex chromosomes. We defined each chromosome as one block, the ESE sites on the chromosome as element sites, and the control sites as flanking sites (for numbers of hit and control positions, see Supplemental Table S1). The analysis was then run roughly in accordance with the procedure used in Gronau et al. (2013) (for full details, see Supplemental Text S1 and Supplemental Table S6).

### multiDFE

The multiDFE program was downloaded from https://bitbucket.org/a_kousathanas/multidfe (last accessed June 14, 2017). SNP data were obtained and filtered as described above and used to calculate site frequency spectra for fourfold degenerate sites overlapping either hit or control positions. multiDFEest was run assuming a series of different distributions (lognormal, gamma, beta, spike, and step models with two to five spikes/steps and the fixed six-spikes model). All models were run assuming either a constant population size or a change in population size (parameter *conpop* set to zero or one). For the step and spike models, 10 repetitions were performed. *fold_SFS* was always set to True. Following the method of Kousathanas and Keightley (2013), the log-likelihood returned by MultiDFE was converted into Akaike's an information criterion value (*AIC* = 2*k* − 2*logl*, where *k* is the parameter number and *logl* is the log likelihood), with a difference of two units in AIC considered as significant. If the best-fit model was a two-steps model and the second best fit within two AIC units was a two-spikes model, the two-spikes model was considered instead, as it is easier to visualize. In order to calculate *N_e_s* values*,* the selective coefficients returned by the program were multiplied by the weighted population size *Nw*.

### Software availability

Custom code used for analysis can be found in the Supplemental Material (Supplemental_Code.zip) or downloaded from https://github.com/rosinaSav/DFE_paper_repo.

## Supplementary Material

Supplemental Material
