# Supplementary material for: Exonic splice regulation imposes strong selection at synonymous sites
Source: Genome Res. 2018 Oct;28(10):1442–54. doi: 10.1101/gr.233999.117 (PMC6169883; doi:10.1101/gr.233999.117)
Supplement: Supplemental Material [file supp_28_10_1442__index.html]

Exonic splice regulation imposes strong selection at synonymous sites — Supplemental Material 

# Exonic splice regulation imposes strong selection at synonymous sites

## Supplemental Material

- Supplemental\_Code.zip
- Supplemental\_Figs\_and\_Texts.pdf
- Supplemental\_Tables.xlsx
