# Supplementary material for: Exonic splice regulation imposes strong selection at synonymous sites
Source: Genome Res. 2018 Oct;28(10):1442–54. doi: 10.1101/gr.233999.117 (PMC6169883; doi:10.1101/gr.233999.117)
Supplement: Supplemental Material [file supp_gr.233999.117_Supplemental_Figs_and_Texts.pdf]

Supplemental data

1. Supplemental Figure 1: Distribution of negative control values for normalized d_S. Supplemental Figures 2, 3 and 4 are included in Supplemental Texts 3 and 5.
2. Supplemental Text 1: Supplemental Methods.
3. Supplemental Text 2: Context-dependent mutational biases.
4. Supplemental Text 3: ESE degeneracy.
5. Supplemental Text 4: Additional analyses (INSIGHT).
6. Supplemental Text 5: Additional analyses (multiDFE).
7. Supplemental Text 6: Additional analyses (normalized d_S).
8. Supplemental Tables found in Supplemental_Tables.xlsx (Supplemental Table 20 can be found in the present document)
    1. Supplemental Table 1: Number of ESE and control sites per chromosome, as used for INSIGHT (INT3).
    2. Supplemental Table 2: Nucleotide composition comparison between ESE and control sites, optimization-based method (INT3).
    3. Supplemental Table 3: Normalized d_S negative control (INT3).
    4. Supplemental Table 4: INSIGHT negative control (INT3).
    5. Supplemental Table 5: Full INSIGHT results (INT3).
    6. Supplemental Table 6: The effect of varying the threshold between high and low frequency minor alleles on the INSIGHT analysis (human filtering; INT3).
    7. Supplemental Table 7: multiDFE negative control (INT3).
    8. Supplemental Table 8: Full multiDFE results (INT3).
    9. Supplemental Table 9: Normalized d_S negative control (other motif sets).
    10. Supplemental Table 10: INSIGHT negative control (ESR).
    11. Supplemental Table 11: INSIGHT negative control (RESCUE).
    12. Supplemental Table 12: INSIGHT negative control (PESE).
    13. Supplemental Table 13: INSIGHT negative control (Ke).
    14. Supplemental Table 14: INSIGHT negative control (combined).
    15. Supplemental Table 15: Full INSIGHT results (other motif sets).
    16. Supplemental Table 16: multiDFE negative control, ancestral *CpG* filtering (other motif sets).
    17. Supplemental Table 17: multiDFE negative control, human *CpG* filtering (other motif sets).
    18. Supplemental Table 18: Full multiDFE results (other motif sets).
    19. Supplemental Table 19: Full multiDFE results (RBP motifs).

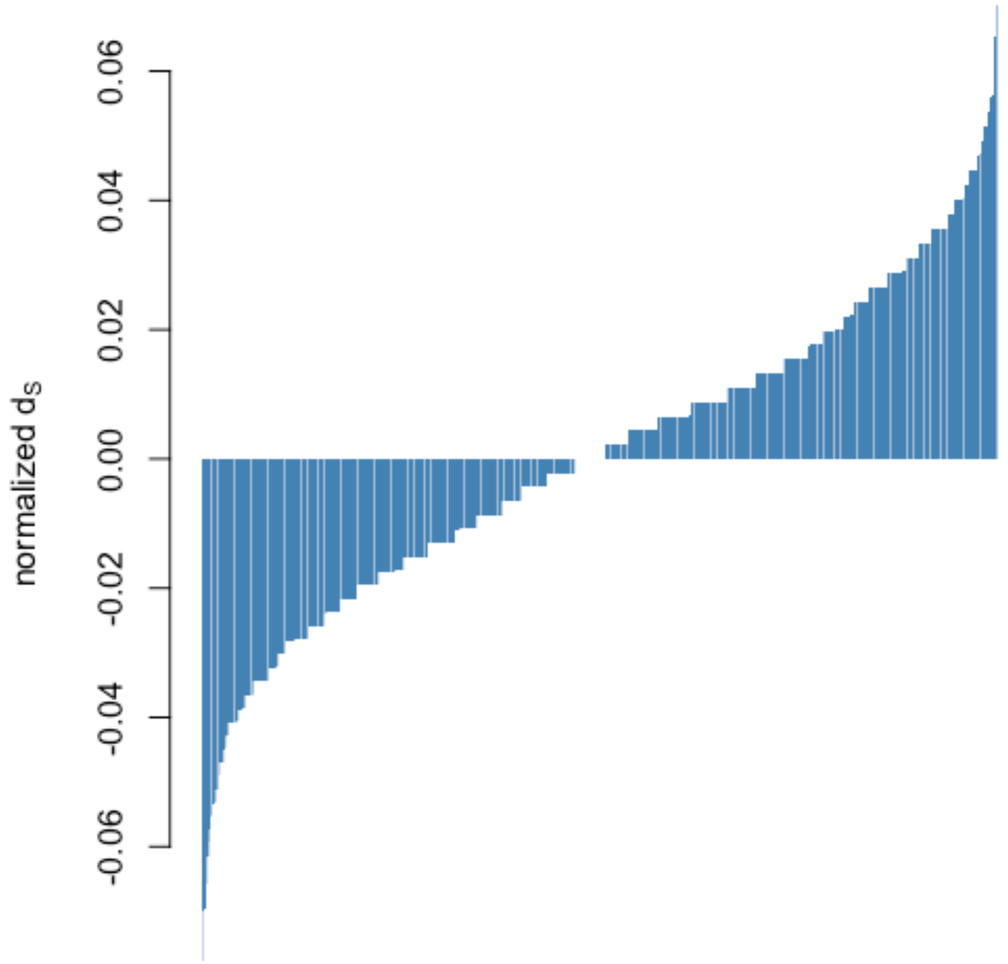

Supplemental Figure 1: Distribution of negative control values for normalized d_S. The values have been sorted from most negative to most positive.

Supplemental Text 1: Supplemental Methods.

Extracting core and flank sequences

Exon core and flank sequences were obtained from the CDSs that were used for the rest of the analysis. The procedure for extracting the sequence regions used for comparing the rate of evolution of Ke motifs in exon flanks and cores can be found in the Materials and Methods section in (Savisaar and Hurst 2017) (although note that in that publication, Ensembl release 78 was used rather than 85). The set of 5' flank sequences, that was used in analyses where exon cores were not taken into consideration, was extracted similarly, except that the minimum exon length was 142 bp, rather than 211.

INSIGHT

As discussed in the main Methods, we defined each chromosome as one block, the ESE sites on the chromosome as element sites and the control sites as flanking sites. We then used a custom Perl script to fetch the 8-primate EPO multiple sequence alignments for the corresponding CDSs from a local installation of release 85 of the Ensembl Compara (Herrero et al. 2016) database and API. Only *Homo sapiens*, *Pan troglodytes*, *Pongo abelii* and *Macaca mulatta* sequences were retained. CDSs where a full-length alignment was not present for all four species were discarded (see Supplemental Table S1 for numbers of remaining sites). Two phylip files were then prepared. The first contained a concatenation of the 4-species multiple sequence alignment for both ESE and control sites combined, with the human sequence replaced with *N*-bases. This file was given as input to the phyloFit programme (Hubisz et al. 2011; Siepel and Haussler 2004) to calculate the conditional probabilities for the nucleotide at each of the ESE/control sites in the human-chimpanzee most recent common ancestor (with the flags *--post-probs*, *--no-rates*, *--no-freqs* turned on, with *subst-mod* set to *JC69* and *init-model* downloaded from ftp://hgdownload.cse.ucsc.edu/goldenPath/hg38/phastCons100way/hg38.phastCons100way.mod (Last accessed: 14 February 2017)). The second phylip file contained a concatenation of control site nucleotides alone. Human sequence was left intact. This file was used to determine the block-specific neutral scaling factor for divergence (Gronau et al. 2013), again using phyloFit (with the flag *--scale-only* turned on, with *subst-mod* set to *JC69* and *init-model* as above). Finally, we calculated the block-specific neutral polymorphism rate as specified in the supplement to Gronau et al. (2013).

The data was formatted as INSIGHT (Gronau et al. 2013) input files and input into runINSIGHT-EM.sh (INSIGHT v.1.1., downloaded from http://compgen.cshl.edu/INSIGHT; last accessed: 9 December 2016) using a L/H frequency cut-off of 15% and default values for all parameters (choosing a different L/H cut-off has little effect on the results (Supplemental Table S6)).

Note that the false positive rates that we obtain with INSIGHT (see main text, notably Figure 1) tend to be higher than those reported in Gronau et al. (2013). This is probably due to the fact that we are using a smaller and hence noisier dataset. As a safety measure, we have therefore not used likelihood ratio tests (LRTs) to evaluate the significance of INSIGHT estimates (unless explicitly indicated), as was done in the original paper. We have rather calculated an empirical one-tailed *p*-value based on where the LRT statistic (twice the log-likelihood difference between the full model and a model where the relevant statistic has been fixed at 0) falls in the distribution of equivalent statistics obtained in the negative control described in the main text ($p = \frac{n+1}{m+1}$, where n is the number of simulants presenting a statistic as high or higher than the true value and m is the number of simulants).

Controlling for nucleotide composition biases

We use different methods of picking control positions for the two model-based methods (INSIGHT and multiDFE) analysis and for everything else. This is because for INSIGHT and multiDFE, it is better to have as many control sites as possible so as to be able to estimate neutral parameters with confidence. With normalized d_S and the comparison of polymorphism frequencies/MAF, on the other hand, it is preferable to match numbers of focal and control sites.

With both methods, we start by dividing the fourfold degenerate sites in our sequences into two groups depending on whether they overlap an ESE motif (*motif sites* and *non-motif sites*). Sites that overlap (certain) *CpG*-dinucleotides are removed from both groups (see below for details). Within each CDS (the full CDS, that is to say, from the start to the stop codon), we then pick a subset of sites from among the non-motif sites in such a way as to match their mononucleotide composition to that of the ESE fourfold degenerate sites. In the equal site numbers strategy, this is achieved simply by sampling with replacement from the non-motif sites to pick the same number of each base as in the set of motif sites (except in the unlikely case that a particular base appears solely among the motif sites, in which case, obviously, that base will have a frequency of 0 among the control sites picked from that CDS).

In the second strategy, used for INSIGHT and multiDFE, we aim not only to match the nucleotide composition of the hit and control sites (though only roughly in this case) but also to obtain as many control sites as possible. To do this, we use an optimization strategy to decide how many sites of each nucleotide to include. Specifically, we use the *optimize.basinhopping()* function, method *L-BFGS-B* from the *scipy* 0.14.1 Python library (Jones et al. 2001) with 500 iterations and a step size of 10. The error function to minimize is the absolute difference between the control nucleotide frequencies and the motif site nucleotide frequencies. Note that the optimizer returns floats, which we round to the nearest integer when evaluating the error function.

We do not explicitly penalize runs that pick low numbers of sites but we promote the inclusion of many sites by choosing large initial guesses. Concretely, we rank the four nucleotides based on how frequent they are at hit sites in a given CDS. For the most frequent nucleotide we set the initial guess to the total number of occurrences of that base among the non-motif sites. For the second most frequent, we set it to three quarters of its frequency at non-motif sites. For the third most frequent, we set it to a quarter of its frequency at non-motif sites. Finally, for the least frequent we set it to 0. Such a strategy was found to be successful both for matching well the nucleotide composition and for obtaining a large number of control sites. Once we have decided on how many of each nucleotide to pick for the control sites in a particular CDS, we select the sites by sampling without replacement from among the non-motif sites.

Supplemental Text 2: Context-dependent mutational biases.

Mutational biases are known to depend on the identity of the adjacent bases (Hess et al. 1994). The method that we use for controlling for nucleotide composition does not account for this fact. We therefore also implemented a second control method. For all non-ESE fourfold degenerate positions within our coding sequences (CDSs), we recorded their trinucleotide context, that is to say, the identity of the nucleotide itself, as well as of the immediately preceding and following nucleotide (no *CpG*-filtering was performed). For each fourfold degenerate ESE position, we then sampled with replacement a control position that had the same trinucleotide context. Note that with the mononucleotide-based control, we always sampled the control site from the same CDS as the hit site, whereas with the trinucleotide-based method, we sampled from across all our CDSs.

We then calculated normalized d_S within INT3 motifs using this new control. The effect was drastic: normalized d_S increased from ≈ -0.273 (normalized d_S obtained with the mononucleotide method with no *CpG*-filtering) to ≈ -0.099 (hit d_S: ≈ 0.054; control d_S: ≈ 0.060). This could mean that the much greater conservation observed using the mononucleotide-based method is really just due to our failure to account for di- or trinucleotide-level mutational biases. However, an alternative explanation is that by requiring not just one but three nucleotides that match the hit position, we are enriching for control sites that are part of motifs that are very similar to the ESE motifs on our list. Many such motifs might actually have ESE activity and have perhaps been left off our list of motifs in error.

In order to see whether this error could be affecting our results, we located all fourfold degenerate positions that were part of a hexamer that differed from an INT3 motif only by a single substitution (*neighbour sites*). We then picked control sites once again but this time did not allow them to overlap neighbour sites. With this new set of control sites, we obtained a normalized d_S of ≈ -0.171 (hit d_S: ≈ 0.054; control d_S: ≈ 0.065). Our best guess at the moment is therefore that the mononucleotide-based method is over-estimating the extent of conservation within ESEs by (very roughly) a third.

However, this result was obtained without filtering out *CpG*s. We therefore repeated the trinucleotide control but this time excluded ancestral *CpG*s, as in the main text. This resulted in a normalized d_S of ≈ -0.080 (hit d_S: ≈ 0.038; control d_S: ≈ 0.041). As before, we then proceeded to also exclude neighbour sites. The result was a truly baffling normalized d_S of ≈ -0.715 (hit d_S: ≈ 0.038; control d_S: ≈ 0.133). We hypothesized that this very high value could simply be due to a large proportion of potential control sites being excluded (almost 60% of potential control positions are removed), resulting in sampling from a very limited population. We therefore performed a simulation where on each of 50 runs, we randomly replaced each neighbour site with a randomly sampled non-ESE fourfold degenerate position. We then repeated the normalized d_S analysis excluding pseudo-neighbour sites from the set of possible control sites. The

resulting distribution of normalized d_S values was centred around a mean of ≈−0.292, with a range from ≈−0.326 to ≈−0.255. It therefore seems that simply removing sites cannot recreate the very high normalized d_S value obtained with the true data. This suggests that it is specifically the removal of neighbour sites that leads to this effect.

Despite strenuous efforts, we have failed to understand why trinucleotide-based sampling with removal of neighbour sites leads to such a high normalized d_S value once ancestral *CpG* positions have been removed. Given the consistency of the results presented in the rest of the manuscript, as well as earlier publications on ESE conservation, this result appears unlikely. It is therefore probably due to some sort of a methodological issue that we have not been able to discover. We therefore conclude that the findings from the trinucleotide control are ambiguous. However, the results obtained without *CpG*-filtering nevertheless suggest that a di- or trinucleotide-level sequence bias might indeed be skewing the results reported in the main text, perhaps causing normalized d_S to be under-estimated by about a third.

Supplemental Text 3: ESE degeneracy.

As discussed in the main text, a potential complication to the analysis is ESE degeneracy – a given substitution may simply exchange one ESE for another. If these two ESEs are functionally interchangeable, then there should be no more selection against the new variant than against a neutral variant at a non-functional site. We would therefore be classifying a functional ESE position as non-functional. This problem could potentially mislead all of the analyses performed in this paper. In divergence-based analyses, it might decrease the difference in substitution rate between hits and controls. In polymorphism-based analyses, it could make us underestimate decreases in polymorphic site frequency and in MAF at ESE sites (because some of the alleles observed segregating at functional sites would be neutral).

In order to overcome this issue, we re-encoded our data so as to only include substitutions/polymorphisms that were *ESE-disruptive*, that is to say, that turned an ESE into a non-ESE. Concretely, we checked all human-macaque differences at fourfold degenerate ESE positions in human. If the change was not ESE-disruptive (i.e. it simply swapped one ESE for another), we changed the macaque allele so that it was identical to the human allele. If this introduced a premature stop codon into the macaque sequence, we replaced both the human and the macaque codon with hyphens so as to discard the codon. For INSIGHT, we proceeded similarly and re-encoded every ESE site allele in the alignment that differed from the human allele as the human allele if the difference was not ESE disruptive. For polymorphism-based analyses, we discarded all SNPs that were not ESE-disruptive.

In order to generate a control, we first scanned all fourfold degenerate ESE positions (independently of whether or not they were divergent) and checked which of the three possible substitutions at that site would be ESE-disruptive. From this, we could calculate a probability that any particular nucleotide

substitution (for instance, a change from an *A* to a *T*) would be ESE-disruptive. For each control site, we then randomly assigned each of the three possible substitutions as either disruptive or not disruptive, with the probability of being assigned as disruptive identical to the probability of such a change being disruptive at true ESE sites. We then modified control data in the same way as we had modified the true data, leaving only those divergences/polymorphisms that had been assigned as disruptive.

As discussed in the main text, the degeneracy-based filtering of divergences had a detectable but small effect on the relative divergence rate within INT3 set ESEs compared to control sites (the same was true for the combined motif set, see main text). We also performed INSIGHT using the degeneracy-filtered data and similarly found relatively little difference when compared to the original dataset (Supplemental Figure 2).

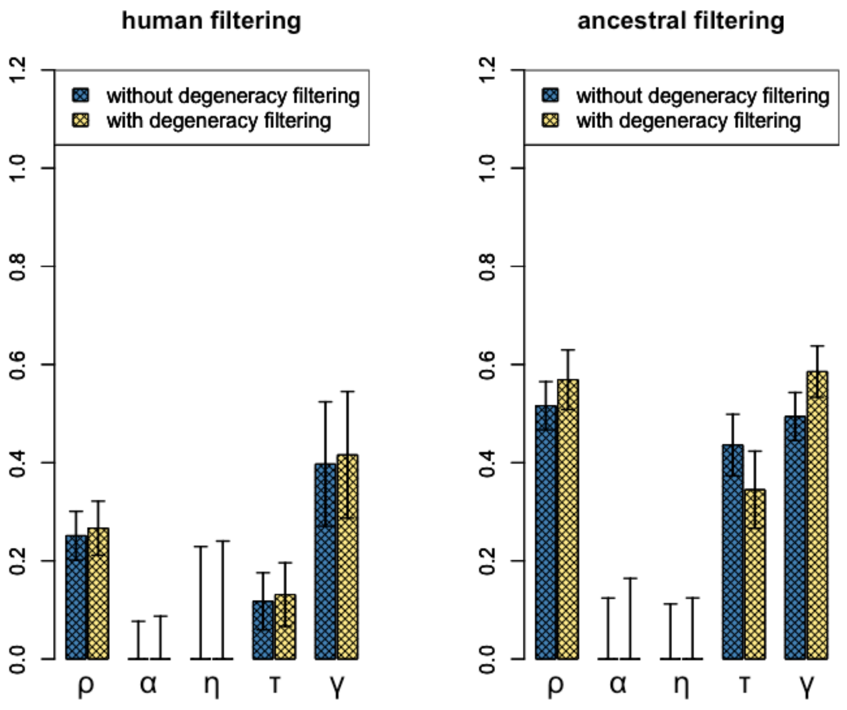

Supplemental Figure 2: Filtering out non-degenerate substitutions/polymorphisms has little effect on INSIGHT results. See legend to Figure 2 in the main text for explanation of symbols.

Relative polymorphism frequencies (compared to control) and MAF were also largely unaffected (Supplemental Table 20).

	polymorphic sites/total sites (hits)	polymorphic sites/total sites (controls)	χ^2 (p)	p from MWU test
4-fold degenerate ESE sites (degeneracy-filtering)	1951/104679 (≈0.019)	2300/104532 (≈0.022)	≈55.079 (≈$1.158 * 10^{-13}$)	≈0.050
4-fold degenerate ESE sites (no degeneracy-filtering)	2364/104679 (≈0.023)	2762/104532 (≈0.026)	≈59.979 (≈$9.589 * 10^{-15}$)	≈0.039

Supplemental Table 20: Frequency of polymorphic sites and minor allele frequencies (MAF) within and outside ESEs, with degeneracy-filtered and non-degeneracy-filtered data. Only human *CpG*-filtering results are shown.

Finally, we also repeated the multiDFE analysis with degeneracy-filtered data and, once again, observed only slight differences compared to non-degeneracy-filtered data (Supplemental Figure 3). The same was true when we used the combined motif set.

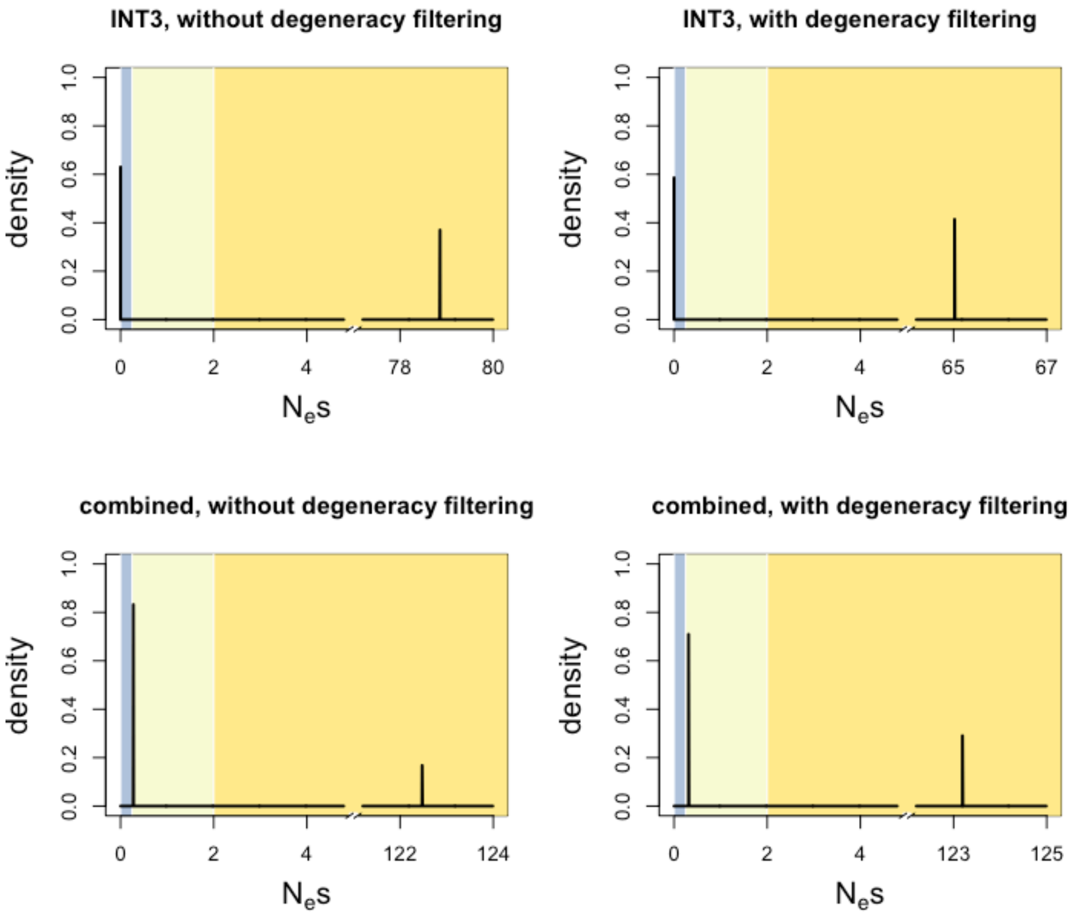

Supplemental Figure 3: Distribution of fitness effects (DFE) for INT3/combined set motifs with either degeneracy-filtered or non-degeneracy-filtered data. Only ancestral *CpG*-filtering based results are shown.

Supplemental Text 4: Additional analyses (INSIGHT).

Positive control

The goal of performing a positive control for the INSIGHT analysis was to make sure that we were able to detect negative selection to preserve amino acid sequence. We aimed to conduct this analysis with data that was as similar as possible to the data used in the ESE analysis in terms of site numbers, nucleotide composition and method of picking controls. We therefore focused exclusively on non-fourfold degenerate sites that overlapped with ESEs. We then picked roughly nucleotide-matched control positions from among non-ESE fourfold degenerate positions (similarly to the procedure used in the main INSIGHT analysis). All *CpG* filtering methods gave similar results, indicating about 80% of sites to be under selection. There was significant evidence for weak negative selection ($p \approx 0$ from LRT), with around 60% of polymorphisms predicted to be under weak negative selection (Figure 2.A in main text). Unlike in previous studies (e.g. Boyko et al. 2008; Bustamante et al. 2005), we found no evidence for positive selection. This might be due to the fact that we only considered positions within ESEs, where the amino acid content appears to be more constrained than elsewhere (Parmley et al. 2006). In other respects, our results

are roughly similar to those from previous studies of selection at non-synonymous sites (Boyko et al. 2008; Eyre-Walker et al. 2006; Keightley and Eyre-Walker 2007; Racimo and Schraiber 2014; Yampolsky et al. 2005). These results confirm that the low site numbers and the protocol used for picking control sites are not preventing INSIGHT from detecting high levels of negative selection.

Controlling for the use of different gene sets in human and ancestral filtering
In theory, any differences in results obtained using ancestral or human filtering could be due to the use of different sets of genes. In order to determine which sites are ancestrally *CpG*, genes are filtered to only leave those that have a complete primate multiple sequence alignment throughout the CDS. Only the most conserved genes are therefore considered. We repeated the analysis of fourfold degenerate ESE sites using human filtering but this time only considered those genes that were included in the ancestral filtering analysis. This led to the removal of ≈48.9% of the genes. The results obtained were nearly identical to those obtained with the full set of genes ($\rho \approx 0.268$; $\tau \approx 0.137$; $\alpha \approx 0.001$). It is therefore unlikely that the differences between filtering methods are simply a result of the use of different gene sets.

Supplemental Text 5: Additional analyses (multiDFE).

Negative control and comparison of CpG-filtering methods
We performed a negative control where we randomly shuffled hits and controls over 100 iterations and ran multiDFE on the shuffled data (Supplemental Table S7). Rather than try out all the possible models and pick the best fit, as for real data, we used the same fixed model in all iterations, namely a beta model with a change in population size. Our statistic was the value of the cumulative distribution function (CDF) of the estimated DFE at $N_e s = 0.1$. multiDFE should not be detecting selection in the negative controls. Hence, this number should be very close to 1 over most runs. To our surprise, ancestral *CpG* filtering achieved a substantially lower false positive rate than human filtering. With human filtering, only 15% of runs exhibited more than 80% of the density below $N_e s = 0.1$, whereas this percentage was 71% for the ancestral filtering (Figure 5 in main text, leftmost plot). We also performed a two-tailed Mann-Whitney *U*-test to compare the CDF(0.1) values of the DFE and found them to be significantly higher with ancestral filtering ($p \approx 4.524 * 10^{-9}$).

Could the lower false positive rate obtained with ancestral filtering be due to the use of more conserved genes (see Supplemental Text 4 for more details)? We repeated the simulation with human filtering but only included genes that were included in the ancestral filtering. The resulting smaller gene set indeed led to a lower false positive rate (33% of runs with more than 80% of the density below $N_e s = 0.1$). However, CDF(0.1) values were still significantly higher with ancestral filtering ($p \approx 0.006$; two-tailed Mann-Whitney *U*-test). It therefore appears that it is genuinely the method of *CpG*-filtering rather than the choice of genes that is leading to the lower false positive rate with ancestral filtering. A possible explanation is that sites that were *CpG* in the human-macaque MRCA are more likely to have undergone a recent substitution than other sites (because they are

hypermutable). A substitution would have momentarily wiped out variation at the site. Any SNPs that are observed in the modern population would therefore necessarily be recent and thus rare, potentially giving the impression of weak purifying selection in sequences that have more ancestral *CpG* than controls.

The effect of failing to account for demography

In all of the multiDFE analyses reported in this manuscript, by far the best predictor of the goodness of model fit (based on Akaike's An Information Criterion (AIC)) was whether or not we allowed for a change in population size (e.g. Supplemental Table S8). We repeated the negative control with ancestral *CpG*-filtering but this time performed each simulation twice, once assuming population size change and once assuming a constant population size. As can be seen in Supplemental Figure 4, failing to account for a change in population size leads to a dramatic increase in the false positive rate. When we assumed a constant population size, not a single negative control run located the majority of the density below N_es = 0.1 (p-value ≈ 2.786 * 10^{-13} from two-tailed Wilcoxon signed-rank test comparing CDF values at N_es = 0.1 in the two conditions). Accounting for changes in population size is therefore crucial, and has fortunately become routine in analyses of this kind (Boyko et al. 2008; Eyre-Walker et al. 2006; Lawrie et al. 2013; Tataru et al. 2017).

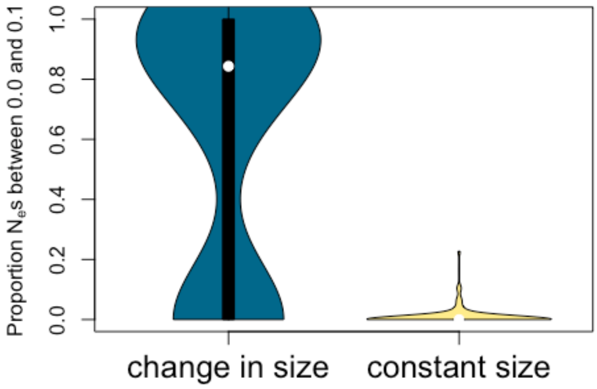

Supplemental Figure 4: multiDFE was run 100 times. Each time, motif hit and control positions were shuffled within each gene, abolishing systematic differences in selective constraints. A large proportion of the density should therefore be below N_es = 0.1 on such a run. This is the case on most runs if one allows for a step change in population size in the past. However, if a constant population size is assumed, multiDFE systematically reports selection where it should be reporting effective neutrality.

Positive control

For the positive control, we analysed non-fourfold degenerate ESE sites with non-ESE fourfold degenerate sites as control (identically to the positive control reported for INSIGHT in Supplemental Text 4; see also Figure 3 in main text). With all *CpG*-filtering methods, the best-fit model (based on AIC) was a two-spike model with a step change in population size in the past (all models were run both under the assumption of a constant population size and allowing for size

change in the past). All *CpG*-filtering methods reported either about a half or about two thirds of incoming mutations to be under strong negative selection. With human filtering or when no filtering was performed, the remainder were found to be under very weak negative selection. With ancestral filtering, the smaller peak was located at $N_e s \approx 0.226$ – right next to our (fairly arbitrary) boundary for distinguishing between effective neutrality and very weak negative selection (at $N_e s = 0.25$). Given the tendency of human filtering to over-estimate the amount of very weak negative selection, the most likely interpretation of the results is that the majority of incoming mutations are strongly deleterious, whilst a substantial minority are either effectively neutral or very weakly deleterious. The latter two groups are teased apart better if we run a three-spikes model rather than a two-spikes one (although this is a slightly worse fit to the data: AIC = 56957.73 *vs* 56961.73). multiDFE now finds a smaller peak (≈8.23% of the density) at $N_e s \approx 1.322 * 10^{-7}$ and a larger peak (≈25.0% of the density) at $N_e s \approx$ 0.300. Reassuringly, our multiDFE setup therefore appears capable of detecting both very weak negative selection and strong negative selection (although note the risk for false positive signals of very weak negative selection that was uncovered through the negative control analysis).

Other CpG-*filtering methods*

In the main text, we have only detailed multiDFE results for ancestral filtering. Here, we briefly report the results for the main analysis (using fourfold degenerate ESE sites as focal sites and fourfold degenerate non-ESE sites as control sites) for all three methods. The best-fit model was a two-spikes model for ancestral and human *CpG* filtering, and a beta model when no *CpG*-filtering was performed, with population size change in the past. In both the no filtering and the ancestral filtering cases, multiDFE indicated about 63% of the density to lie within effective neutrality and about 37% of mutations to be under very strong negative selection (with $N_e s$ roughly around 100; Figure 6 in main text, top left plot; Supplemental Table S8). Human filtering estimated the first peak to be larger (≈83.3%) and located within very weak negative selection ($N_e s \approx 0.535$). However, given the results of the negative control, we have relied on the findings obtained with ancestral filtering in the main text.

Supplemental Text 6: Additional analyses (normalized d_S).

Additional analysis on Ke motifs

Theoretically, the signal of fast evolution detected at Ke motif sites could be an artefact due to sampling motif sites from fast-evolving exon cores, where Ke motifs are more frequent (Cáceres and Hurst 2013), and control sites from exon flanks. This does not seem to be the case: the signal remains when we restrict the analysis to 5' exon flanks (hit $d_S \approx 0.054$; control $d_S \approx 0.044$; normalized $d_S \approx 0.230$) or to exon cores (hit $d_S \approx 0.055$; control $d_S \approx 0.042$; normalized $d_S \approx 0.290$) alone.

We can also ask whether this signal of fast evolution is specific to an unusual subset of the motifs, with perhaps the remainder of the set showing more expected patterns. The Ke motifs were determined fully experimentally, by introducing all possible hexamers at several locations within two different reporter minigenes (Ke et al. 2011). Certain hexamers enhanced splicing mainly

in the exon flanks, whilst others were more active in the core. Given that ESEs are usually thought to be more common and potentially also more active in the exon flanks (Cáceres and Hurst 2013; Woolfe et al. 2010; Wu et al. 2005), could it be that the fast evolution is primarily a property of those (perhaps more atypical) Ke ESEs that appear to have a stronger splice-enhancing effect in the exon core? We filtered the full set of motifs to leave only those that were more active when placed in the 5' exon flank than when placed in the exon core (see *Methods* for more details). This left us with a reduced set of 95 motifs. We then repeated the normalized d_S analysis using this set alone. The signal of positive selection not only remained but also grew stronger (hit $d_S \approx 0.071$; control $d_S \approx 0.051$; normalized $d_S \approx 0.372$), suggesting that it is not due to an atypical subset of ESEs that are more active in the exon core than the flank.

Normalized d_S *for the combined set, excluding ESR.*

A potential caveat of the analyses performed on the combined set of motifs is that one of the component sets (ESR) was partially defined by looking for conserved motifs. This renders our analysis somewhat circular. We therefore recalculated normalized d_S but this time excluded those motifs that only appeared in ESR (this left 2298 motifs). This more conservative analysis leads to qualitatively similar results to the full analysis (hit $d_S \approx 0.043$; control $d_S \approx 0.056$; normalized $d_S \approx -0.240$). It is thus unlikely that the inclusion of this set of motifs substantially biases our results.

Boyko AR et al. (2008) Assessing the evolutionary impact of amino acid mutations in the human genome. PLoS Genet 4:e1000083

Bustamante CD et al. (2005) Natural selection on protein-coding genes in the human genome. Nature 437:1153-1157

Cáceres EF, Hurst LD (2013) The evolution, impact and properties of exonic splice enhancers. Genome biology 14:1–18

Eyre-Walker A, Woolfit M, Phelps T (2006) The distribution of fitness effects of new deleterious amino acid mutations in humans. Genetics 173:891-900

Gronau I, Arbiza L, Mohammed J, Siepel A (2013) Inference of natural selection from interspersed genomic elements based on polymorphism and divergence. Mol Biol Evol 30:1159-1171

Herrero J et al. (2016) Ensembl comparative genomics resources. Database : the journal of biological databases and curation 2016:1-17

Hess ST, Blake JD, Blake RD (1994) Wide Variations in Neighbor-dependent Substitution Rates. J Mol Biol 236:1022-1033

Hubisz MJ, Pollard KS, Siepel A (2011) PHAST and RPHAST: phylogenetic analysis with space/time models. Brief Bioinform 12:41-51

Jones E, Oliphant T, Peterson P, et al. (2001) SciPy: Open Source Scientific Tools for Python.

Ke S et al. (2011) Quantitative evaluation of all hexamers as exonic splicing elements. Genome Res 21:1360-1374

Keightley PD, Eyre-Walker A (2007) Joint inference of the distribution of fitness effects of deleterious mutations and population demography based on nucleotide polymorphism frequencies. Genetics 177:2251-2261

Lawrie DS, Messer PW, Hershberg R, Petrov DA (2013) Strong purifying selection at synonymous sites in D. melanogaster. PLoS Genet 9:e1003527

Parmley JL, Chamary JV, Hurst LD (2006) Evidence for Purifying Selection Against Synonymous Mutations in Mammalian Exonic Splicing Enhancers. Molecular Biology and Evolution 23:301-309

Racimo F, Schraiber JG (2014) Approximation to the distribution of fitness effects across functional categories in human segregating polymorphisms. PLoS Genet 10:e1004697

Savisaar R, Hurst LD (2017) Both maintenance and avoidance of RNA-binding protein interactions constrain coding sequence evolution. Mol Biol Evol

Siepel A, Haussler D (2004) Phylogenetic estimation of context-dependent substitution rates by maximum likelihood. Mol Biol Evol 21:468-488

Tataru P, Mollion M, Glemin S, Bataillon T (2017) Inference of Distribution of Fitness Effects and Proportion of Adaptive Substitutions from Polymorphism Data. Genetics 207:1103-1119

Woolfe A, Mullikin JC, Elnitski L (2010) Genomic features defining exonic variants that modulate splicing. Genome Biol 11:R20

Wu Y, Zhang Y, Zhang J (2005) Distribution of exonic splicing enhancer elements in human genes. Genomics 86:329-336

Yampolsky LY, Kondrashov FA, Kondrashov AS (2005) Distribution of the strength of selection against amino acid replacements in human proteins. Hum Mol Genet 14:3191-3201
